# Extracellular Calcium Dictates Onset, Severity, and Recovery of Diarrhea in a Child with Immune-Mediated Enteropathy

**DOI:** 10.3389/fped.2018.00007

**Published:** 2018-01-29

**Authors:** Johnathan Fraebel, Regino Gonzalez-Peralta, Maryann Maximos, Genie L. Beasley, Christopher Douglas Jolley, Sam Xianjun Cheng

**Affiliations:** ^1^Department of Pediatrics, Gastroenterology, Hepatology, and Nutrition, University of Florida, Gainesville, FL, United States

**Keywords:** calcium, calcium metabolism, calcium-sensing receptor, diarrhea, immune-mediated enteropathy, inflammatory bowel disease, intestinal barrier function, ion transport

## Abstract

Diarrhea causes monovalent and divalent ion losses that can influence clinical outcome. Unlike the losses of monovalent ions, such as Na^+^, K^+^, Cl^−^, and ${\text{HCO}}_{3}^{-}$, which are generally large in quantity (osmoles) and therefore determine the severity of diarrhea, the losses of divalent ions are relatively small in osmoles and are often overlooked during diarrheal treatment. Studies now suggest that despite divalent ions being small in osmoles, their effects are large due to the presence of divalent ion-sensing receptors and their amplifying effects in the gut. As a result, losses of these divalent ions without prompt replacement could also significantly affect the onset, severity, and/or recovery of diarrheal disease. Herein, we report a case of a malnourished child with an immune-mediated enteropathy who developed episodes of “breakthrough” diarrhea with concurrent hypocalcemia while on appropriate immunotherapy. Interestingly, during these periods of diarrhea, stool volume fluctuated with levels of blood Ca^2+^. When Ca^2+^ was low, diarrhea occurred; when Ca^2+^ levels normalized with replacement, diarrhea stopped. Based on this and other observations, a broader question arises as to whether the Ca^2+^ lost in diarrhea should be replaced promptly in these patients.

## Background

Diarrhea causes both monovalent and divalent ion losses (1, 2). Without prompt replacement, both affect the outcome of diarrheal disease. While losses of monovalent ions Na^+^, K^+^, Cl^−^, and ${\text{HCO}}_{3}^{-}$ are large and therefore determine the severity of diarrhea, losses of divalent ions are small but large in biologic effect due to the presence of divalent ion-sensing receptor-mediated signal amplification (3, 4). As a result, without prompt replacement, the loss of divalent ions can also likely affect the onset, severity, and recovery of diarrheal disease. For example, Zn^2+^, acting via Zn^2+^-sensing receptor (ZnSR), can reduce the severity, duration, and recurrence rate of diarrhea (5). This Zn^2+^ effect is particularly important in mal- and undernourished children in whom an underlying negative balance of Zn^2+^ metabolism often exists. However, despite the recent advances in Ca^2+^ and Ca^2+^-sensing receptor (CaSR) research, limited information is available on the role and function of this important divalent ion in diarrhea.

In this communication, we report a malnourished child with an immune-mediated enteropathy. Despite adequate immunotherapy, he developed episodes of “breakthrough” diarrhea with concurrent hypocalcemia. Interestingly, during these disease flare-up episodes, diarrhea symptoms inversely fluctuated with levels of blood Ca^2+^. When blood (serum) Ca^2+^ was low, diarrhea occurred; when Ca^2+^ levels normalized with replacement, diarrhea quickly stopped. In light of this and other observations, we propose that, similar to Zn^2+^ deficiency, the loss of Ca^2+^ without prompt replacement may compromise the diarrhea-protective capability of Ca^2+^ and CaSR in the gut and lead to severe, protracted, and recurrent diarrhea.

## Case Presentation

**Figure 1:**
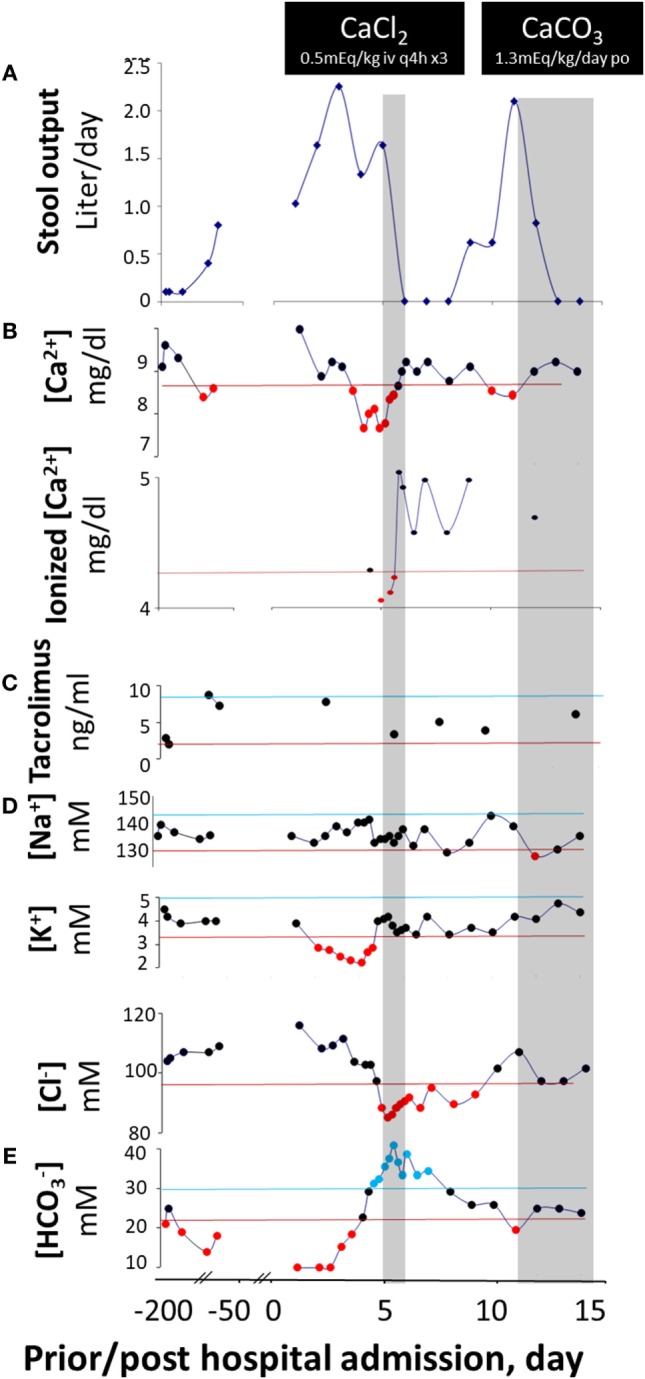
Diarrhea response and fluctuation of laboratory values prior to and post calcium replacement. Stool output **(A)** does not vary with blood levels of tacrolimus **(C)** but fluctuates with blood concentrations of Ca^2+^ ([Ca^2+^]) **(B)** on three occasions and via two routes of administration. Upon switch to a dairy-free diet, [Ca^2+^] reduces and diarrhea occurs. With intravenous CaCl_2_, [Ca^2+^] rises and stool output declines. With oral CaCO_3_, [Ca^2+^] rises again and stool output yet declines again. The corresponding alterations in other laboratory values are shown in **(D,E)**. The values between the upper lines (upper normal limits) and lower lines (lower normal limits) are normal, whereas the values above the upper lines are elevated and those below the lower lines are reduced. The two gray areas illustrate the periods in which the patient was receiving calcium therapies. The form of calcium salts and their doses administered are indicated in the black boxes. It is likely that under a negative status of calcium balance, the diarrhea-combating ability of the divalent mineral is compromised, and the responsiveness to immunomodulators is lost. As a result, disease is “flared up” and the patient becomes diarrheic. Diarrhea will temporarily be halted upon normalization of calcium level. However, the disease will relapse and will not enter a permanent remission state unless a normal positive calcium balance is restored.

The patient is a 6-year-old African-American male with autoimmune enteropathy (diagnosed 12 months prior to admission, based on the following criteria: intractable diarrhea, small bowel villous atrophy, presence of circulating anti-enterocyte antibodies, and responsiveness to immunosuppressive treatment) who was hospitalized for worsening non-bloody, watery diarrhea, severe malnutrition, and hypocalcemia. Initially, he responded well to glucocorticosteroid monotherapy. However, for the 6 months prior to this admission, social issues gradually led to interruption of this therapy with recurrence of diarrhea (Figure 1A) and weight loss. Despite re-initiation of glucocorticoids, diarrhea persisted, so tacrolimus was added to his treatment regimen. He responded well with combination therapy, but 6 weeks prior to admission, he developed recurrent diarrhea and weight loss (despite appropriate administration of medications and consistent therapeutic tacrolimus levels). He had no other medical problems and had no relatives with gastrointestinal or immunological disease. Findings on the initial physical examination revealed a moderately malnourished (height *Z* = −0.56, weight *Z* = −2.25, BMI *Z* = −2.85) child with hypothermia (temperature 97.5 ^°^F), hypotension (blood pressure 83/54 mmHg), tachycardia (pulse 103 beats per minute), dry mucus membranes, sunken orbits, temporal wasting, and a protuberant but otherwise benign abdomen. His laboratory values are shown in Figures 1B–E.

He was initially resuscitated with intravenous fluids. However, his diarrhea (Figure 1A) and metabolic acidosis (Figure 1E) remained while hypokalemia (Figure 1D) worsened. All enteral intakes were withheld and total parenteral nutrition was initiated, while intravenous solumedrol and oral tacrolimus were continued. Despite good therapeutic blood levels of tacrolimus (Figure 1C), the diarrhea persisted (Figure 1A). Pan cultures of stool, urine, and blood revealed no abnormal growth. Stool ova and parasites analysis, viral, and *Clostridium difficile* toxin A/B studies were negative. On hospital day 4, he developed severe hypocalcemia with tetany (contractures of the hands and lower extremities), and worsening hypokalemia and metabolic acidosis. He was transferred to the intensive care unit for a closer monitoring and given three consecutive doses of q 3-h intravenous calcium chloride (0.5 mEq elemental Ca^2+^/kg/dose), in addition to intravenous fluids and other electrolyte replacements. Remarkably, as his serum-ionized Ca^2+^ normalized, his diarrhea resolved (Figure 1A). In fact, after 3 days of calcium therapy, he became constipated.

With weaning of calcium supplementation, there was a recurrence of diarrhea, hypocalcemia, and metabolic acidosis. Five days after discontinuing calcium, his daily stool output increased to more than 2 L—close to that before calcium supplementation. The diarrhea resolved within 2 days of administration of oral calcium carbonate suspension (1.3 mEq elemental Ca^2+^/kg/day) (Figure 1A). Of note, he received a combination of glucocorticoid-tacrolimus (at therapeutic levels) therapy during the entire hospitalization. At discharge, the patient was prescribed vitamin D in addition to calcium supplementation to restore normal Ca^2+^ balance. He was also placed on an unrestricted diet. At his recent 3-month follow-up clinic visit, he had no diarrhea, and the serum calcium levels remained normal.

## Discussion

Management of pediatric diarrhea remains challenging, particularly in children with malnutrition or undernutrition, in whom diarrheal episodes are often severe, protracted, and recurrent. Based on the previous Ca^2+^ metabolic balance studies in diarrhea (1, 2) and the recent work on the influence of Ca^2+^ and CaSR in reversing both secretory (6–10) and inflammatory diarrheas (11–13), we propose that the inadequate replacement of Ca^2+^ losses and the resultant inadequate activation of intestinal CaSR may be responsible for the severity and persistence of diarrhea symptoms in these malnourished patients.

According to earlier metabolic balance studies (1, 2), diarrhea results in losses of not only Na^+^, K^+^, Cl^−^, and ${\text{HCO}}_{3}^{-}$, which are normally replaced with oral rehydration solution (ORS), but also Ca^2+^, Mg^2+^, and Zn^2+^, which are not routinely included in these solutions, possibly because of their relatively small quantities compared to monovalent ion losses (see summary in Table 1).

**Table 1:** Total fluid, electrolyte, and mineral losses in normal infants and those with diarrhea and their consequences.

Loss	Normal	With diarrhea	Fold increase	Consequences	
Total	84	171	2.0		
H_2_O	81	162	2.0	Hypovolemia	↑ Mortality
Na^+^	2.15	12.35	5.7	Hypovolemia	
Cl^−^	2.69	9.36	3.5	Hypovolemia	
K^+^	4.15	4.92	1.2	Hypokalemia	
${\text{HCO}}_{3}^{-}$	3.61	7.91	2.2	Metabolic acidosis	

Ca^2+^	0.16	3.27	20.5	Ca^2+^ deficiency → ↓CaSR	↑ Morbidity
Mg^2+^	0.30	0.83	2.8	Mg^2+^ deficiency → ↓CaSR	
Zn^2+^	0.00072	0.00243	3.4	Zn^2+^ deficiency → ↓ZnSR	

*The total water loss is expressed in gram/kg body weight/day, while electrolyte losses and mineral losses are in milli-mole/kg body weight/day. The calculation of Zn^2+^ loss is based on the data obtained from a metabolic balance study in human infants (2), whereas the calculations of other losses are based on the data from a metabolic balance study in infant calves (1). The loss of $HCO_{3}^{-}$ is estimated by subtracting the Cl^−^ loss from the combined Na^+^ and K^+^ losses. Different from monovalent ions, divalent ions have additional effects on physiologic processes besides their established nutritional functions. These effects include the activation of extracellular calcium-sensing receptor (CaSR) by Ca^2+^ and Mg^2+^ and the activation of extracellular zinc-sensing receptor (ZnSR) in the intestine (refer to text for details)*.

It is important to note that the absolute concentrations of divalent ion losses in diarrhea are smaller than those of monovalent ones, and they contribute less to the severity of dehydration. However, divalent mineral losses may greatly affect the course of diarrhea, leading to prolonged duration and frequent recurrence of the disease. Unlike monovalent ions, divalent ions such as Ca^2+^ are “functional” nutrients. In addition to their nutritional values, they also function as hormones or first messengers, binding to their corresponding divalent ion-sensing receptors (i.e., Ca^2+^ and Mg^2+^ binding to CaSR (3, 4, 13) and Zn^2+^ binding to ZnSR (5)) in the enterocytes of the gastrointestinal tract and inhibiting pathophysiologic processes that result in diarrhea. Indeed, as shown in this present case, diarrhea volume inversely correlated with serum levels of Ca^2+^ (Figure 1). Similar findings were also observed in children with infectious diarrheal diseases (14), as well as in healthy adult volunteers who were infected with enterotoxigenic *Escherichia coli* resulting in secretory diarrhea (15).

**Figure 2:**
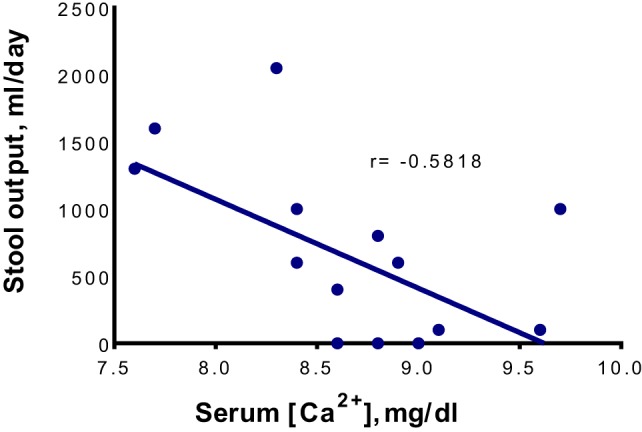
The correlation between serum Ca^2+^ concentrations ([Ca^2+^]) and daily stool outputs. The total serum [Ca^2+^] is shown. The linear regression was performed using Microsoft Excel 2016 for Windows, while the statistical analysis was performed using GraphPad Prism version 6.07 for Windows (GraphPad Software, San Diego, CA, USA). The fluctuation in stool output is correlated significantly with the change in total (*r* = −0.5818, *P* = 0.0181) and ionized (*r* = −0.8396, *P* = 0.0365, data not shown) serum [Ca^2+^] in a negative manner.

Considering the chronic nature of the diarrhea prior to admission and the absence of gastrointestinal infection, our patient’s diarrhea was most likely related to the underlying autoimmune enteropathy. Indeed, the diarrhea responded well to appropriate immunotherapy while calcium levels were normal. However, it recurred with hypocalcemia and again resolved with normalization of this element’s serum levels. The direct correlation between calcium concentration and stool volume (Figure 2) strongly implicates hypocalcemia as the cause of “breakthrough” diarrhea in this patient. The fact that the intensification of immunosuppression alone (with low calcium levels) early during his hospitalization failed to curtail diarrhea also lends support to this conclusion.

There are multiple reasons that our patient would have a negative balance of Ca^2+^ metabolism. First, he has malnutrition. It is common for children with malnutrition to have a negative calcium balance. Second, this child had a history of enteropathy that could impair Ca^2+^ absorption and induce Ca^2+^ loss. Third, he also has milk-dairy intolerance and was consuming a dairy-free diet. The long restriction of Ca^2+^-rich dairy intake could lead his Ca^2+^ metabolic balance to a further negative direction, precipitating hypocalcemia and diarrhea.

The mechanisms whereby hypocalcemia precipitates diarrhea in an inflammatory gut have been described in animal models and not in humans. Several studies have reported that Ca^2+^ is required for the maintenance of epithelial tight junction integrity, a critical determinant of intestinal barrier function and inflammatory diarrhea (16–25). Decreases in extracellular Ca^2+^ concentration caused cell–cell junction destruction and opening of the paracellular pathway (17, 18, 20–22, 25). As a result, animals on high Ca^2+^ diets were shown to be more resistant to the development of enterocolitis caused by barrier function disrupters, such as invading pathogens (26–29), chemical colitogens (30), and immune-mediated processes (31). By contrast, animals on reduced Ca^2+^ diets were more prone to induced intestinal inflammation, resulting in colitis and diarrhea (32). Emerging data are now linking this phenomenon to the activity of the CaSR, as activation of this special G-protein-coupled cell surface receptor increased the epithelial tight junction barrier (12, 25) while its inactivation [as in CaSR-deficient mice (11)] disrupted intestinal barrier, leading to increased gut inflammation. In these CaSR-deficient mice, diarrhea responses to induced gut inflammation were more severe and persisted longer than their wild-type littermates. Based on these experimental observations, we propose that Ca^2+^-therapeutic effects on inflammatory diarrhea are mediated via the CaSR, augmenting the epithelial barrier function that is so often disrupted in these conditions.

Of note, the localization of CaSR in the gut has been described on both the apical and the basolateral sides of the plasma membrane of the enterocyte (8), and receptors in both membrane domains of the polarized enterocyte are functionally active and can be activated by Ca^2+^, calcimimetics such as R568, and other polycations such as spermine with similar potency and EC_50_ values (7–9). It is therefore possible that the efficacy of oral Ca^2+^ supplementation results from the activation of the CaSR at the intestinal brush border, whereas the efficacy of intravenous Ca^2+^ supplementation results from the activation of the receptor at the blood side of the enterocyte. These observations demonstrate that the anti-diarrheal activity of Ca^2+^ can be readily achieved by either route. Thus, depending upon the severity of symptoms and the presence or absence of emergencies, the replacement of Ca^2+^ can be given either intravenously or orally, as in the case of this patient. However, it is worth noting that, while the repletion via intravenous route raises serum Ca^2+^ quickly, this quickly raised serum calcium is lost quickly due to its prompt activation of CaSR in parathyroid glands and kidneys (which increases Ca^2+^ excretion). By contrast, while oral replacement raises serum Ca^2+^ relatively slowly, it produces local therapeutic anti-diarrheal (and other) actions and helps restore Ca^2+^ balance without causing much unwanted systemic adverse effects. Given this, the oral route is considered both safer and more physiological. Whenever clinically allowed, prompt switching from intravenous to oral supplementation of Ca^2+^ is recommended.

## Concluding Remarks

Given the wealth of accumulating data on the role and importance of Ca^2+^ and CaSR in both secretory and inflammatory diarrheal conditions, we suggest that Ca^2^ stool losses should be routinely replaced as is currently done for Na^+^, K^+^, Cl^−^, ${\text{HCO}}_{3}^{-}$, and Zn^2+^. As summarized in Table 2, an ideal ORS composition would contain both monovalent ions and divalent minerals. The monovalent ions aim at replacing electrolyte losses, and correcting both hypovolemia and metabolic acidosis, thereby reducing diarrhea-associated mortality. By contrast, divalent ions would replace mineral losses, restoring the anti-diarrheal activities of CaSR and ZnSR, and thereby reducing the onset, duration, and recurrence of diarrhea. ORS use has been declining over the past decade, with fewer than 33% of children with diarrhea under the age of 5 using this therapy (33). Because it has little effects in decreasing stool volume, caregivers are reluctant to use ORS and instead prefer using anti-microbial agents, which increases the risk of developing drug resistance (3). If the findings presented in this and other studies are confirmed, adding divalent minerals to, or concurrently supplementing these ions along with ORS, would likely increase compliance with this oral therapy. Large randomized-controlled trials are warranted to further test the efficacy and safety of this therapeutic strategy.

**Table 2:** Rationale for suggested combined approach for treating diarrhea.

	Function	Outcome	
ORS	Replace Na^+^, K^+^, Cl^−^, and ${\text{HCO}}_{3}^{-}$ losses	Correct hypovolemia, hypokalemia, and metabolic acidosis	↓Mortality
Zn^2+^ supplementation	Replace Zn^2+^ loss	Increase ZnSR	↓Morbidity
Ca^2+^/Mg^2+^ supplementation	Replace Ca^2+^/Mg^2+^ loss	Increase CaSR	

*ORS, oral rehydration solution; ZnSR, Zn^2+^-sensing receptor; CaSR, Ca^2+^-sensing receptor*.

## Consent for Publication

The written consent for publication was obtained from the child’s parent.

## Ethics Statement

This study was carried out in accordance with the recommendations of the University of Florida Health Center’s IRB Guidelines and Privacy Rules about case reports, and it has been reviewed by UF IRB with written informed consent from the guardian of the studied subject.

## Author Contributions

SXC conceptualized and designed the work, JF and SXC collected and analyzed the data, RG, MM, GLB, CDJ, and SXC interpreted the data, JF and SXC drafted and RG, MM, GLB, and CDJ revised this manuscript, and all authors approved the final version and agreed to be accountable for the content of this work.

## Conflict of Interest Statement

The authors declare that the submitted work was conducted in the absence of any commercial or financial relationships that could be construed as a potential conflict of interest.

## References

[1] Lewis LD Phillips RW Water and electrolyte losses in neonatal calves with acute diarrhea. A complete balance study. Cornell Vet (1972) 62(4):596–607. 5077538

[2] Castillo-Duran C Vial P Uauy R. Trace mineral balance during acute diarrhea in infants. J Pediatr (1988) 113(3):452–7. 10.1016/S0022-3476(88)80627-9 3411389

[3] Cheng SX. Calcium-sensing receptor: a new target for therapy of diarrhea. World J Gastroenterol (2016) 22(9):2711–24. 10.3748/wjg.v22.i9.2711 26973410 PMC4777994

[4] Tang L Cheng CY Sun X Pedicone AJ Mohamadzadeh M Cheng SX. The extracellular calcium-sensing receptor in the intestine: evidence for regulation of colonic absorption, secretion, motility, and immunity. Front Physiol (2016) 7:245. 10.3389/fphys.2016.00245 27458380 PMC4914593

[5] Sunuwar L Asraf H Donowitz M Sekler I Hershfinkel M. The Zn2+-sensing receptor, ZnR/GPR39, upregulates colonocytic Cl- absorption, via basolateral KCC1, and reduces fluid loss. Biochim Biophys Acta (2017) 1863(4):947–60. 10.1016/j.bbadis.2017.01.009 28093242 PMC5557417

[6] Cheng SX. Calcium-sensing receptor inhibits secretagogue-induced electrolyte secretion by intestine via the enteric nervous system. Am J Physiol (2012) 303(1):G60–70. 10.1152/ajpgi.00425.2011 22517767 PMC3404579

[7] Cheng SX Geibel J Hebert S. Extracellular polyamines regulate fluid secretion in rat colonic crypts via the extracellular calcium-sensing receptor. Gastroenterology (2004) 126(1):148–58. 10.1053/j.gastro.2003.10.064 14699496

[8] Cheng SX Okuda M Hall A Geibel JP Hebert SC. Expression of calcium-sensing receptor in rat colonic epithelium: evidence for modulation of fluid secretion. Am J Physiol (2002) 283:G240–50. 10.1152/ajpgi.00500.2001 12065312

[9] Geibel J Sritharan K Geibel R Geibel P Persing JS Seeger A Calcium-sensing receptor abrogates secretagogue-induced increases in intestinal net fluid secretion by enhancing cyclic nucleotide destruction. Proc Natl Acad Sci U S A (2006) 103(25):9390–7. 10.1073/pnas.0602996103 16760252 PMC1475505

[10] Tang L Peng M Liu L Chang W Binder HJ Cheng SX Calcium-sensing receptor stimulates Cl – and SCFA-dependent but inhibits cAMP-dependent HCO3- secretion in colon. Am J Physiol (2015) 308(10):G874–83. 10.1152/ajpgi.00341.2014 PMC4437021 25792563

[11] Cheng SX Lightfoot YL Yang T Zadeh M Tang L Sahay B Epithelial CaSR deficiency alters intestinal integrity and promotes proinflammatory immune responses. FEBS Lett (2014) 588(22):4158–66. 10.1016/j.febslet.2014.05.007 24842610 PMC4234694

[12] Winesett S Tang L Shi J Cheng SX Calcium-sensing receptor regulation of integrity of tight junction and intestinal barrier: role of nutrients. Gastroenterology (2017) 152(5 Suppl 1):S765 10.1016/S0016-5085(17)32656-2

[13] Owen JL Cheng SX Ge Y Sahay B Mohamadzadeh M. The role of the calcium-sensing receptor in gastrointestinal inflammation. Semin Cell Dev Biol (2016) 49:44–51. 10.1016/j.semcdb.2015.10.040 26709005 PMC4761506

[14] Cheng SX Bai HX Gonzalez-Peralta R Mistry PK Gorelick FS. Calcium ameliorates diarrhea in immunocompromised children. J Pediatr Gastro Nutr (2013) 56(6):641–4. 10.1097/MPG.0b013e3182868946 23343935 PMC4448079

[15] Bovee-Oudenhoven IMJ Lettink-Wissink MLG Van Doesburg W Witteman BJM Van Der Meer R. Diarrhea caused by enterotoxigenic *Escherichia coli* infection of humans is inhibited by dietary calcium. Gastroenterology (2003) 125(2):469–76. 10.1016/S0016-5085(03)00884-9 12891550

[16] Cereijido M Meza I Martinez-Palomo A. Occluding junctions in cultured epithelial monolayers. Am J Physiol (1981) 240(3):C96–102. 10.1152/ajpcell.1981.240.3.C96 7212057

[17] Sedar AW Forte JG. Effects of calcium depletion on the junctional complex between oxyntic cells of gastric glands. J Cell Biol (1964) 22:173–88. 10.1083/jcb.22.1.173 14195608 PMC2106493

[18] Hays RM Singer B Malamed S. The effect of calcium withdrawal on the structure and function of the toad bladder. J Cell Biol (1965) 25(3 Suppl):195–208. 10.1083/jcb.25.3.195 5840797 PMC2106690

[19] Galli P Brenna A Camilli de P Meldolesi J Extracellular calcium and the organization of tight junctions in pancreatic acinar cells. Exp Cell Res (1976) 99(1):178–83. 10.1016/0014-4827(76)90694-7 816663

[20] Meldolesi J Castiglioni G Parma R Nassivera N De Camilli P. Ca++-dependent disassembly and reassembly of occluding junctions in guinea pig pancreatic acinar cells. Effect of drugs. J Cell Biol (1978) 79(1):156–72. 10.1083/jcb.79.1.156 701369 PMC2110211

[21] Palant CE Duffey ME Mookerjee BK Ho S Bentzel CJ. Ca2+ regulation of tight-junction permeability and structure in *Necturus gallbladder*. Am J Physiol (1983) 245(3):C203–12. 10.1152/ajpcell.1983.245.3.C203 6412561

[22] Martinez-Palomo A Meza I Beaty G Cereijido M. Experimental modulation of occluding junctions in a cultured transporting epithelium. J Cell Biol (1980) 87(3 Pt 1):736–45. 10.1083/jcb.87.3.736 6780571 PMC2110771

[23] Gonzalez-Mariscal L Chavez de Ramirez B Cereijido M Tight junction formation in cultured epithelial cells (MDCK). J Mem Biol (1985) 86(2):113–25. 10.1007/BF01870778 4032460

[24] Gong Y Renigunta V Himmerkus N Zhang J Renigunta A Bleich M Claudin-14 regulates renal Ca(+)(+) transport in response to CaSR signalling via a novel microRNA pathway. EMBO J (2012) 31(8):1999–2012. 10.1038/emboj.2012.49 22373575 PMC3343334

[25] Jouret F Wu J Hull M Rajendran V Mayr B Schofl C Activation of the Ca(2)+-sensing receptor induces deposition of tight junction components to the epithelial cell plasma membrane. J Cell Sci (2013) 126(Pt 22):5132–42. 10.1242/jcs.127555 24013548 PMC3828589

[26] Bovee Oudenhoven IM Termont DS Heidt PJ Van der Meer R. Increasing the intestinal resistance of rats to the invasive pathogen Salmonella enteritidis: additive effects of dietary lactulose and calcium. Gut (1997) 40(4):497–504. 10.1136/gut.40.4.497 9176078 PMC1027125

[27] Bovee Oudenhoven IM Wissink ML Wouters JT Van der Meer R. Dietary calcium phosphate stimulates intestinal lactobacilli and decreases the severity of a salmonella infection in rats. J Nutr (1999) 129(3):607–12. 10082763 10.1093/jn/129.3.607

[28] Bovee-Oudenhoven IM Termont DS Weerkamp AH Faassen-Peters MA Van der Meer R. Dietary calcium inhibits the intestinal colonization and translocation of *Salmonella* in rats. Gastroenterology (1997) 113(2):550–7. 10.1053/gast.1997.v113.pm9247475 9247475

[29] Bovee-Oudenhoven I Termont D Dekker R Van der Meer R. Calcium in milk and fermentation by yoghurt bacteria increase the resistance of rats to *Salmonella* infection. Gut (1996) 38(1):59–65. 10.1136/gut.38.1.59 8566860 PMC1382980

[30] Cheng SX Calcium-sensing receptor in the gut: evidence for its role in mediating known nutritional therapy for inflammatory bowel disease. JPGN (2012) 55(Suppl 1):E70.

[31] Schepens MA Vink C Schonewille AJ Dijkstra G van der Meer R Bovee-Oudenhoven IM Supplemental calcium attenuates the colitis-related increase in diarrhea, intestinal permeability, and extracellular matrix breakdown in HLA-B27 transgenic rats. J Nutr (2009) 139:1525–33. 10.3945/jn.109.105205 19535420

[32] Pele LC Thoree V Mustafa F He S Tsaprouni L Punchard NA Low dietary calcium levels modulate mucosal caspase expression and increase disease activity in mice with dextran sulfate sodium induced colitis. J Nutr (2007) 137:2475–80. 17951488 10.1093/jn/137.11.2475

[33] Donowitz M Alpers DH Binder HJ Brewer T Carrington J Grey MJ Translational approaches for pharmacotherapy development for acute diarrhea. Gastroenterology (2012) 142:e1–9. 10.1053/j.gastro.2012.01.014 PMC5881109 22266149

